# A perioperative consult service results in reduction in cost and length of stay for colorectal surgical patients: evidence from a healthcare redesign project

**DOI:** 10.1186/s13741-016-0028-1

**Published:** 2016-02-05

**Authors:** Matthew D. McEvoy, Jonathan P. Wanderer, Adam B. King, Timothy M. Geiger, Vikram Tiwari, Maxim Terekhov, Jesse M. Ehrenfeld, William R. Furman, Lorri A. Lee, Warren S. Sandberg

**Affiliations:** Department of Anesthesiology, Vanderbilt University School of Medicine, Vanderbilt University Hospital, Nashville, TN 37232 USA; Department of Biomedical Informatics, Vanderbilt University School of Medicine, Vanderbilt University Hospital, Nashville, TN 37232 USA; Division of Colon and Rectal Surgery, Vanderbilt University School of Medicine, 1161 21st Ave South, D5248, Nashville, TN 37232-2543 USA; Department of Surgery, Vanderbilt University School of Medicine, Vanderbilt University Hospital, Nashville, TN 37232 USA; Department of Health Policy, Vanderbilt University School of Medicine, Vanderbilt University Hospital, Nashville, TN 37232 USA; Division of Neuroanesthesiology, Vanderbilt University School of Medicine, Vanderbilt University Hospital, Nashville, TN 37232 USA

**Keywords:** Care redesign, Outcomes, Length of stay, Cost, Colorectal surgery, Consult service, Multimodal

## Abstract

**Background:**

A major restructuring of perioperative care delivery is required to reduce cost while improving patient outcomes. In a test implementation of this notion, we developed and implemented a perioperative consult service (PCS) for colorectal surgery patients.

**Methods:**

A 6-month planning process was undertaken to engage key stakeholders from surgery, nursing, and anesthesia in a healthcare redesign project that resulted in the creation of a PCS to implement a coordinated clinical pathway. After Institutional Review Board (IRB) approval, data were collected for all elective colorectal procedures for three phases: phase 0 (pre-implementation; 1/2014–6/2014), phase 1 (7/2014–10/2014), and phase 2 (11/2014–10/2015). Length of stay (primary endpoint; LOS), total hospital cost, use of clinical pathway components, markers of functional recovery, and readmission and reoperation rates were analyzed. Outcomes and patient characteristics among phases were compared by two-tailed *t* tests and Wilcoxon rank-sum tests. Categorical variables were analyzed by chi-square and Fisher’s exact tests.

**Results:**

We studied 544 patients (phase 0 = 179; phase 1 = 124; phase 2 = 241), with 365 consecutive patients being cared for in the redesigned care structure. Median LOS was reduced and sustained after implementation (phase 0, 4.24 days; phase 1, 3.32 days; phase 2, 3.32 days, *P* < 0.01 phase 0 v. phases 1 and 2), and mean LOS was reduced in phase 2 (phase 0, 5.26 days; phase 1, 4.93 days; phase 2, 4.36 days, *P* < 0.01 phase 0 v. phase 2). Total hospital cost was reduced by 17 % (*P* = 0.05, median). Application of clinical pathway components was higher in phases 1 and 2 compared to phase 0 (*P* < 0.01 for all components except anti-emetics); measures of functional recovery improved with successive phases. Reoperation and 30-day readmission rates were no different in phase 1 or phase 2 compared to phase 0 (*P* > 0.15).

**Conclusions:**

Restructuring of perioperative care delivery through the launch of a PCS-reduced LOS and total cost in a significant and sustainable fashion for colorectal surgery patients. Based on the success of this care redesign project, hospital administration is funding expansion to additional services.

**Electronic supplementary material:**

The online version of this article (doi:10.1186/s13741-016-0028-1) contains supplementary material, which is available to authorized users.

## Background

Perioperative care in the USA is often costly and fragmented. A major restructuring is needed to improve care coordination, quality, outcomes, and access, all while restraining or reducing cost (Holt 2014; Huang and Schweitzer 2014; Vetter et al. 2013). The Institute for Healthcare Improvement (IHI) has proposed a Triple Aim, which is to (a) improve the individual experience of surgical care, (b) improve the health of a defined surgical population, and (c) reduce the per capita cost of surgical care (Vetter et al. 2014; Berwick and Whittington 2008). Prior work has demonstrated that careful, multidisciplinary perioperative system redesign can improve operating room throughput (Cendan and Good 2006; Harders et al. 2006; Sandberg et al. 2005; Smith et al. 2008), and the concepts guiding this work are likely scalable to the entire perioperative period. Accordingly, the concept of the Perioperative Surgical Home (PSH), which is a patient-centered, physician-led, interdisciplinary, and team-based system of coordinated care for the procedural and surgical patient, has been proposed as a model of healthcare redesign in the USA (Cannesson and Kain 2014; Schweitzer et al. 2013). However, the actual structure and function of such an entity remain vague.

Additionally, while Enhanced Recovery After Surgery (ERAS) care pathways have been employed for several decades in Europe, few studies in the USA have described the effect of implementing such pathways outside of a strict research setting and within a new model of care that spans the perioperative period, is sustainable, and described in a manner that can be replicated (Persson et al. 2015; Lei et al. 2015; Bryson 2015; Page et al. 2015; Bona et al. 2014; Batdorf et al. 2014; Wang et al. 2015; Miller et al. 2014). It is clear that a protocol itself is not enough (Gillissen et al. 2015; Maessen et al. 2007). It has been proposed that ERAS care pathways are specific tools that can be utilized within a PSH to create a high-reliability, low-variability, coordinated system of perioperative care in a sustainable model (Cannesson and Kain 2014). But, what that entails in practical terms remains unclear, and clear demonstrations of care redesign success are needed.

Accordingly, in partnership with colorectal surgeons, perioperative nurses, and the anesthesia care team at our institution, we launched a perioperative consult service (PCS). The purpose of this service was to implement and sustain highly coordinated care for colorectal surgical (CRS) patients from the time a decision was made to operate through the entire perioperative period, including post-discharge follow-up. We hypothesized that an effective implementation would be temporally associated with an outcome that patients, payors, and hospitals value: faster recovery and reduction in length of stay (LOS) and hospital costs.

## Methods

### Study design

We performed a retrospective, observational (before and after) care redesign study. After approval from the Vanderbilt University Human Research Protection Program (Institutional Review Board (IRB)), perioperative data were collected from medical and billing records for all elective colorectal procedures performed for the 6 months preceding implementation (1/2014–6/2014; phase 0), for 4 months following implementation (7/2014–10/2014; phase 1), and for nine additional months (11/2014–10/2015; phase 2). As part of an ongoing quality improvement project, data were prospectively collected and retrospectively analyzed. As such, the IRB granted a waiver of informed consent. Our primary outcomes of interest were LOS and hospital cost. Secondary outcomes included adherence to ERAS bundle components, functional recovery milestones, and reoperation and readmission rates, as detailed below. We describe the process of redesign below. Of note, no changes were made at the service line or institutional level that would have affected LOS or case and co-morbidity coding during the entire study period of redesign and analysis.

### Process analysis and redesign

Prior to the introduction of the PCS and ERAS pathway, there was no standardization of care in the areas of perioperative pain management, postoperative nausea and vomiting (PONV) prophylaxis, intravenous fluid management, early ambulation, or early re-feeding. In 2012 and 2013, colorectal surgeons at our institution standardized intraoperative care processes (e.g., bowel isolation) to reduce surgical site infection risk and also standardized and stabilized their postoperative care processes to ensure that discharge closely followed functional recovery. Prior to the implementation of our care redesign project, LOS for colorectal surgery at our institution was in the top decile (“Exemplary’” designation) in the National Surgery Quality Improvement Project (NSQIP) (NSQIP data extraction for the pre-implementation period; last accessed September 16, 2015.)

With participation from key stakeholders, a review of the literature was performed and historical data at our institution were reviewed, including resource LOS, hospital costs, anesthetic management, perioperative pain and nausea/vomiting management, readmissions, and reoperations. The initial process started with 6 months of coordinated planning between surgeons, nurses, pharmacists, and anesthesiologists to establish (a) the initial components of the ERAS pathway for colorectal surgical patients, (b) the coordination of daily workflow and decision-making from the preoperative surgical visit and our anesthesia preoperative evaluation clinic (PEC) through all phases of care, including the post-discharge period, and (c) the iterative process of data evaluation and plan-do-study-act (PDSA) cycles using control chart methodology.

### Daily processes

**Fig. 1:**
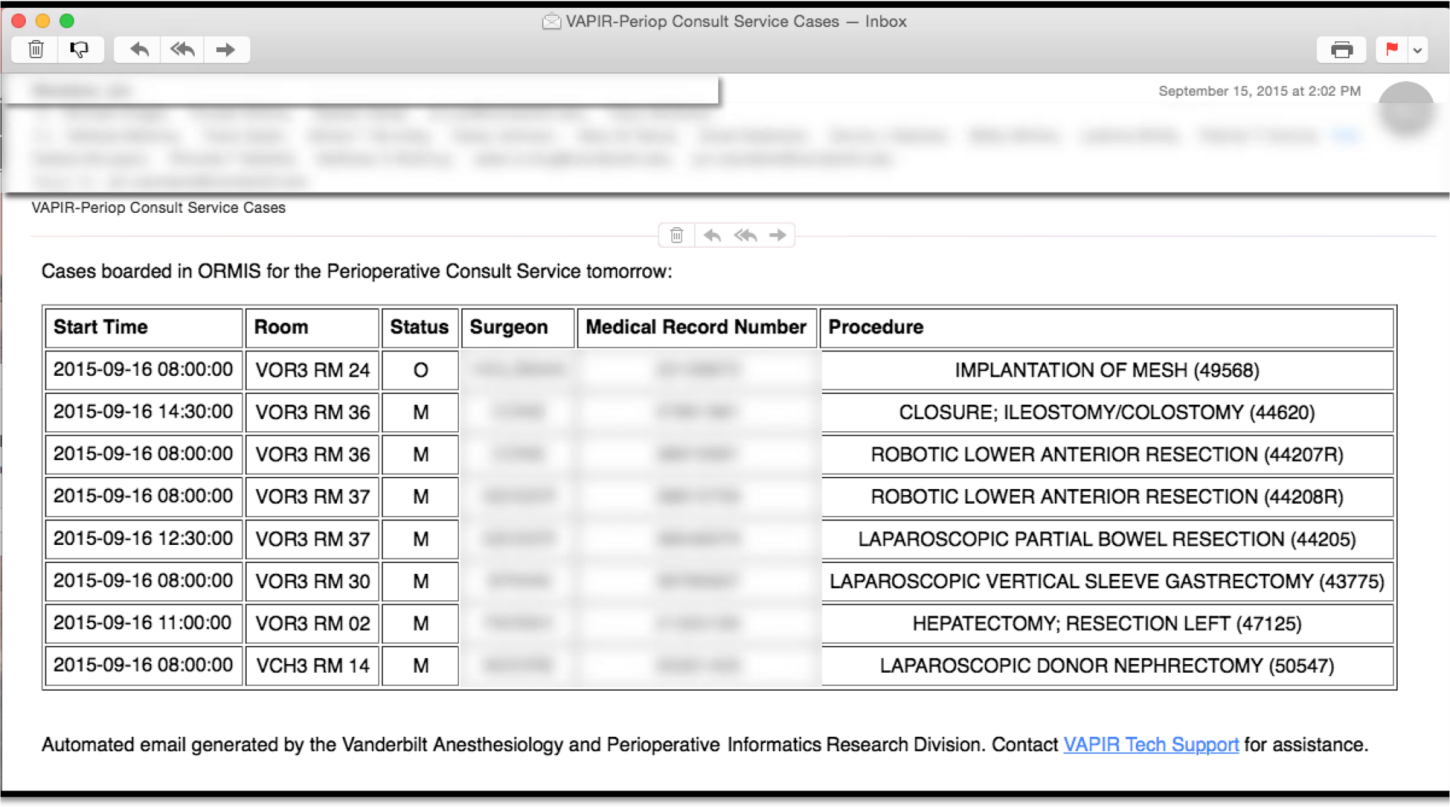
Example of automated daily email based on clinical assignments. This screenshot displays an example of the automated daily email that is sent each day at 3 pm to perioperative team members based on clinical assignments. The case list is generated from programmed logic concerning case type and surgeon. This list is used by perioperative nurses and the PCS to allocate resources to enhanced recovery care pathway patients the following day. As can be seen from this figure, patients from multiple surgical services are cared for by the PCS. *PCS* perioperative consult service]

**Fig. 2:**
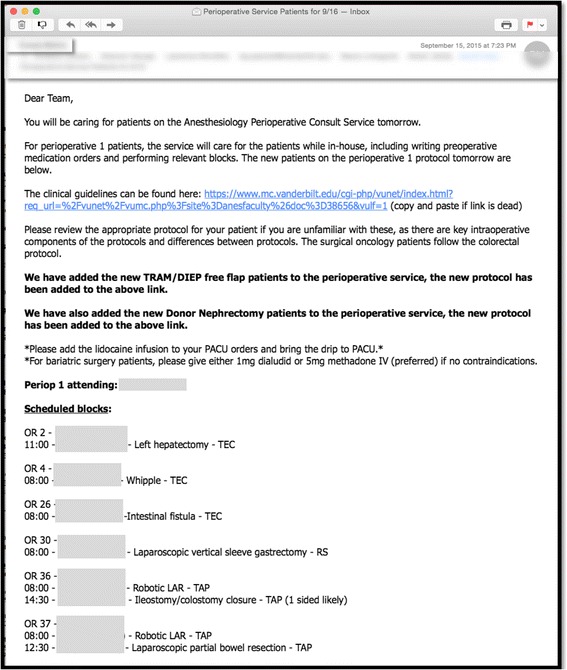
Example of daily email to coordinate implementation of anesthesia components of enhanced recovery care pathways. This screenshot displays an example of a daily email sent by a member of the PCS to the attending anesthesiologists and in-room anesthesia providers in order to coordinate preoperative, intraoperative, and postoperative implementation of the anesthesia components of the enhanced recovery care pathways. As can be seen from this figure, patients from multiple surgical services are cared for by the PCS according to case-specific ERAS pathways. *PCS* perioperative consult service, *ERAS* enhanced recovery after surgery

**Fig. 3:**
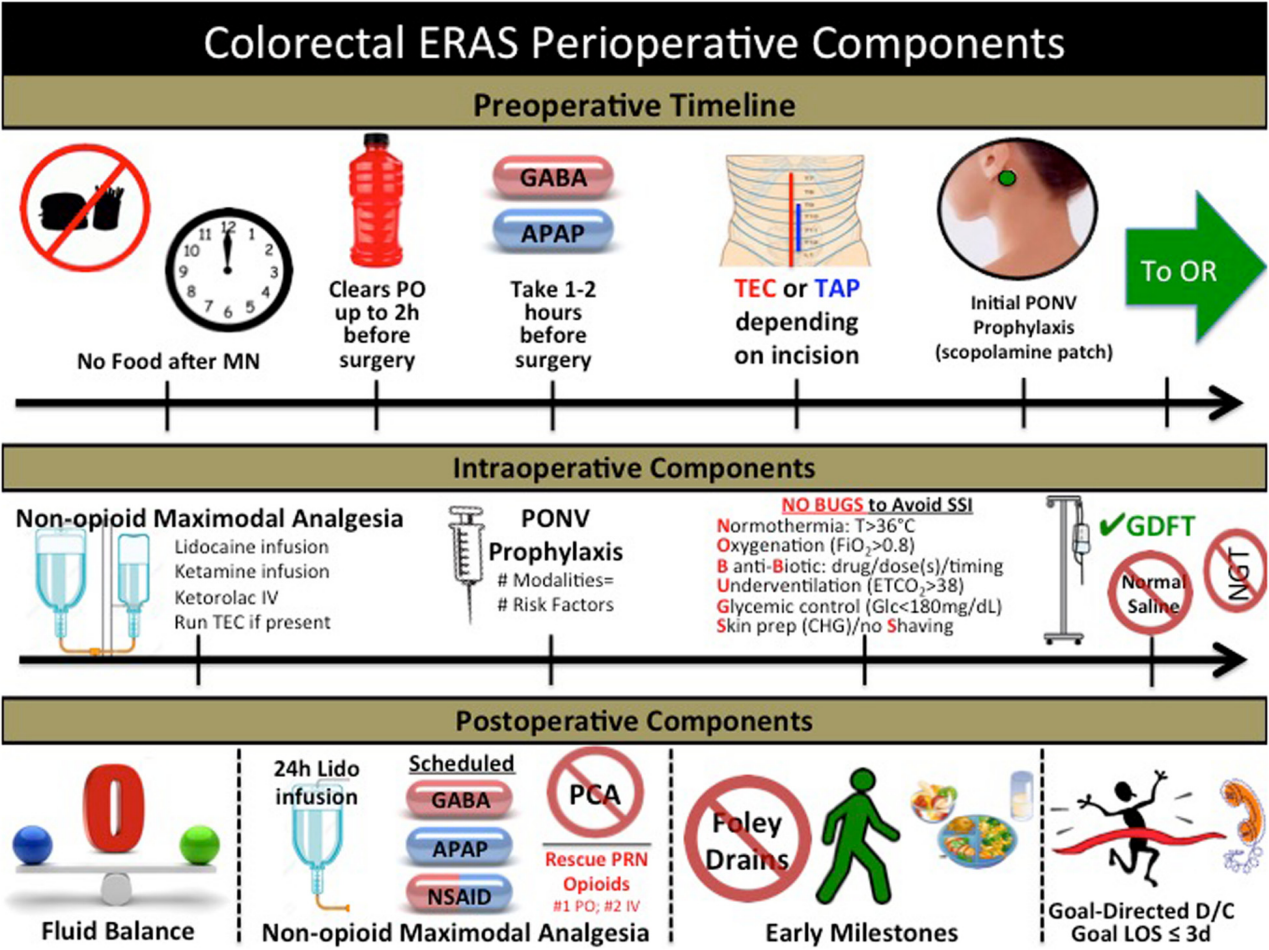
Colorectal ERAS perioperative components. This figure illustrates the principles and goals of the ERAS pathway for colorectal surgical patients at our institution in each phase of care, starting the night before surgery. Of note, the preoperative oral fluid loading on the night before and morning of surgery is currently in the initial implementation phase. *ERAS* enhanced recovery after surgery

This care redesign project consisted of several key components for daily implementation. First, at 3 pm each day, an automated query is run against the operating room (OR) schedule for the next day. The OR cases scheduled that meet specific Current Procedural Terminology (CPT) criteria (see *Cases Included in Analysis* below) are extracted and complied into a tabular form and sent via automated email to the PCS team members, including preoperative nursing staff (see Fig. 1). This list is used for care planning for the next day, including a prioritization of when these patients are brought into the preoperative holding room area. Second, one resident on the team performs a manual review of patient charts and then takes this list of cases and inserts it into a pre-formatted daily email that is sent to all anesthesia providers who will be caring for these patients in the OR (see Fig. 2). This step is performed to ensure that all anesthesia care team members (a) are aware that their patients are to be cared for by the principles of the ERAS care pathway, (b) have a way to access that pathway online, and (c) know the exact components of the ERAS pathway to be applied in each patient, as multimodal pain management, nausea/vomiting prophylaxis, and other components of a care pathway can be altered based upon patient history, age, and medication list (e.g., anticoagulants). This step is in the process of being automated. [Of note, all patients were previously evaluated by our Preoperative Evaluation Clinic (PEC) in order to ensure to proper assessment and management of co-morbidities had occurred. The PEC assessment typically occurs 1–2 weeks prior to surgery. Patients are assessed by our Preoperative Evaluation Clinic through in-person visits and through phone call screening. All patients are then reassessed on the day of surgery by the attending anesthesiologist caring for them that day. Patients that fall into the ASA PS 3 or 4 classification are seen in person, whereas ASA PS 1 and 2 patients are typically screened over the phone and through a review of our EHR. None of these processes changed throughout the study period.] The review of the patient record on the day prior to surgery is to ensure that proper individualization is made in the care pathway, if needed (e.g., proper dosing of gabapentin in the elderly and review of anticoagulant medications and latest coagulation studies in patients receiving a thoracic epidural). Third, on the day of surgery, a morning checkout occurs that reviews the overnight course of the postoperative patients already on the PCS in order to assess whether any interventions are needed prior to addressing new patients that morning. Fourth, premedications are given and first case truncal or neuraxial nerve blocks are performed by the PCS team according to case type and care pathways (see Fig. 3). At this time, as first cases are being taken to the operating room, a standard communication occurs with the surgical team concerning patient progress and disposition, and then floor rounds are completed by the PCS team on all inpatients on service, which is an average daily census of 20 to 25 patients currently. For any patient being discharged that day, a 2-week postoperative pain management and nausea prophylaxis plan is specified by the PCS, and prescriptions are written. Throughout the day, the next round(s) of patients receive premedications and preoperative nerve blocks from the PCS team. Fifth, throughout the day, the senior resident on service and the anesthesiologist receive consults from our nurse practitioner (NP)-led PEC. These cases are discussed, and if needed, a special care plan is constructed for the patient and communicated between the surgeon and anesthesiologist. Sixth, for patients discharged within the preceding 24 h, a member of the PCS team performs a home call to assess continued recovery of function, level of pain and nausea, and understanding of medication regimen. During the month preceding the launch of the ERAS pathway and the PCS, education was disseminated through key stakeholders to their respective teams (nursing, surgery, and anesthesia) concerning these components of care.

After launching the project, we continued frequent meetings between anesthesiologists, surgeons, and nurses and performed iterative plan-do-study-act (PDSA) cycles. During phase 1, this multidisciplinary team discussed areas of potential improvement but did not make any changes to the initial clinical pathway. Two specific changes implemented in phase 2 included the addition of a 24-h postoperative intravenous lidocaine infusion and an increased focus on early re-feeding; the former being based upon medical literature and the latter based upon our ongoing review of our performance (McCarthy et al. 2010). Our process is currently stabilized with phase 2 interventions.

### Cases included in analysis

Four colorectal surgeons at Vanderbilt University Medical Center performed all of the procedures included in this study. We included every elective procedure resulting in an inpatient admission where one of our colorectal surgeons scheduled a case that included one or more specified Current Procedural Terminology codes (44320, 44188, 44310, 44187, 44227, 44312, 44314, 44340, 44345, 44227, 44620, 44120, 44202, 45550, 45402, 45540, 44205, 44160, 44204, 44140, 44207, 44145, 44208, 44146, 44210, 44150, 44211, 44158, 45136, 44212, 44155, 45395, 45110, 4539, 45126, 45119, and 44626). This procedure list consists of colonic resections, rectal resections, ostomy creation, and reversal. We included ostomy patients because ileus is one of the most common causes of prolonged hospital stay after any bowel surgery and because many components of the ERAS pathway are intended to promote early return of bowel function in an effort to decrease LOS in all types of bowel surgery.

### Data collection

We obtained data from our perioperative data warehouse including patient demographics, type of procedure, surgical and anesthesia duration, and information about intraoperative ERAS pathway components (McPherson 2009). Electronic medication administration records were analyzed to determine postoperative medication usage. Usage of patient-controlled analgesia was determined by the analysis of order entry data. The presence of nausea and quantification of pain, postoperative urine output, fluid administration, time to first oral intake, and first stool output were determined by the analysis of nursing flow sheet data. Pain scores were obtained as part of routine nursing care using an 11-point numerical rating scale (0–10), and pain score means were computed for each postoperative day. Readmissions were defined as any inpatient or observation status admission to our institution for any reason within 30 days following the day of discharge. Reoperations were defined as any anesthetic provided at our institution within 30 days following the end of the surgical procedure. Case mix index (CMI), hospital cost, and resource length of stay (LOS) were obtained from hospital administrative data sources. We report total actual costs, which refers to all technical /hospital costs. This includes all fixed and variable costs. As such, it includes direct and indirect labor, direct and indirect supplies, and direct and indirect facilities. We chose to use this metric as total actual costs are the highest level of aggregate costs. Because the comparison of LOS in whole days may miss the subtlety of discharge timing, LOS was measured in fractional days between the earliest time of admission into any hospital unit and the time of physical discharge (resource LOS) in order to assess whether any reduction in LOS would actually correlate with an open bed into which an additional surgical patient could be admitted.

Although some patients included in phase 0 (the baseline group) had received some of the components of the ERAS pathway prior to the launch (e.g., nerve blocks, non-opioid adjuncts), they remained in the pre-intervention group for analysis as our focus was primarily on the new team and process of care delivery.

### Statistical analysis

To track the progress of this project, we created a control chart to monitor LOS performance over time. For the ease of display and interpretation, we used bins of seven cases, which represents the weekly average number of colorectal surgical cases in phase 0. Control charts and related statistical process control methodologies have been adapted to healthcare and used to monitor processes in anesthesiology, where they have the advantage of allowing early detection of process performance changes (Benneyan et al. 2003; Sandberg et al. 2008; Ehrenfeld et al. 2009).

Two-tailed *t* tests and Wilcoxon-Mann-Whitney (Wilcoxon rank-sum) tests were used to compare outcomes and patient characteristics among the phases. Chi-square and Fisher’s exact tests were applied to analyze categorical variables. The Spearman correlation coefficient was calculated to assess the relationship between continuous variables. A two-sided α level of 0.05 was taken as reference to detect statistical significance in all analyses. Statistical programming was implemented in SAS 9.4 (SAS Institute Inc., Cary, NC, USA).

## Results

Five hundred forty-four patients were included in the analysis. There were 179 patients in phase 0, 124 in phase 1, and 241 in phase 2. Analysis of patient demographics and case characteristics among the three groups demonstrated a statistically significant (*P* = 0.02) increase in CMI in phase 1 after instituting the PCS with ERAS, but no change in ASA physical status (Table 1, *P* > 0.05). A complete listing of all comparisons of patient demographics and surgical case information is shown in Table 1.

**Table 1:** Patient demographics and surgical case information

	Phase 0 (*N* = 179)	Phase 1 (*N* = 124)	Phase 2 (*N* = 241)	*P*		
				0 v. 1	1 v. 2	0 v. 2
Age (y)	52 ± 18	49 ± 18	53 ± 17	0.10	0.02	0.68
Gender (F/M)	94/85	64/60	31/38	0.91	0.32	0.45
Height (cm)	171 ± 11	171 ± 10	172 ± 14	0.85	0.12	0.14
Weight (kg)	78 ± 21	79 ± 18	80 ± 20	0.34	0.86	0.15
BMI (kg/m^2^)	26 ± 6	27 ± 6	28 ± 9	0.40	0.92	0.19
ASA physical status						
1 and 2	75 (41.9 %)	62 (50.0 %)	102 (42.3 %)	0.20	0.16	0.93
3 and 4	104 (58.1 %)	62 (50.0 %)	139 (57.7 %)			
Case mix index	2.18 ± 0.93	2.56 ± 1.71	2.32 ± 0.95	0.02	0.06	0.13
Type of surgery						
Resection	138	88	162	0.23	0.47	0.03
Ostomy creation/reversal	41	36	79			
Laparoscopic (%)	56.4 %	51.6 %	46.9 %	0.41	0.39	0.05
Duration of surgery (min)	158 ± 82	162 ± 83	169 ± 90	0.87	0.47	0.25
Duration of anesthesia (min)	204 ± 88	214 ± 90	217 ± 96	0.44	0.73	0.17

Data as mean ± SD for continuous variables

*y* years, *F* female, *M* male, *BMI* body mass index, *ASA* American Society of Anesthesiologists, *min* minutes

Table 2 reports statistical comparisons of the main study outcomes. Median LOS was reduced from 4.24 days during phase 0 to 3.32 days in phase 1 (*P* < 0.01 for phases 0 v. 1), and remained unchanged at 3.32 days in phase 2 (*P* < 0.01 phases 0 v. 2, *P* > 0.05 for phases 1 v. 2). A financial analysis of this program demonstrated a decrease in median total hospital costs per patient by 17 % in phases 2 v. 0 (*P* = 0.05). The 30-day readmission rates and reoperation rates were not significantly different in phase 1 or phase 2 compared to phase 0, though power was very low for this comparison (*P* > 0.05) (Table 2). As such, the combination of reduced LOS and cost reveals that four CRS patients can now be cared for in the same time as three patients in the baseline group at a significantly reduced cost compared to historical baseline. See (Additional file 1) for a complete listing of secondary outcomes, which demonstrates a high rate of compliance with care pathway components and earlier functional recovery after implementation of the care redesign.

**Table 2:** Effect of implementation of major study outcomes

	Phase 0 (*N* = 179)	Phase 1 (*N* = 124)	Phase 2 (*N* = 241)	*P*		
				0 v. 1	1 v. 2	0 v. 2
Mean resource LOS (days)	5.26	4.93	4.36	0.47	0.15	<0.01^a^
Median resource LOS (days)	4.24	3.32	3.32	<0.01^a^	0.61	<0.001^a^
Reoperation	18 (10.1 %)	13 (10.5 %)	15 (6.22 %)	1	0.15	0.20
Readmissions	21 (11.7 %)	18 (14.5 %)	34 (14.1 %)	0.49	0.92	0.48
Hospital cost	100 %	98 %	83 %	0.05^a^		

^**a**^Significant at 5 % level; % non-parametric median test for no difference in median cost among all phases

**Fig. 4:**
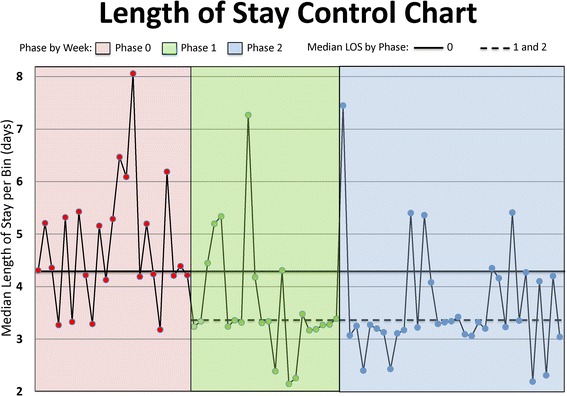
Length of stay control chart. Example of a LOS run chart that contains data that are reviewed each week and at scheduled monthly meetings. Data are presented in groups of seven patients, as this is the average weekly number of colorectal surgery cases performed. Overall median LOS is reduced in phase 1 compared to phase 0 and has remained lower in phase 2. *LOS* length of stay

Figure 4 is a version of the working control chart used to monitor the primary outcome, LOS, during the project, with each point representing the median LOS for approximately 1 week of successive CRS patients, with no exclusions. The median of this control chart (representing characteristic performance in phase 0) was established prior to the initiation of phase 1. As we were evaluating time-series data in a real-world setting, we used control chart methodology to define the bounds of phase 1 (i.e., seven successive points on one side of the historical median). As such, we continued with phase 1 until a signal appeared indicating that a sustained performance shift had occurred and a new median LOS should be calculated. We then calculated a new median, including all (but only) the patients in phase 1 and then initiated phase 2. All gains have been sustained throughout phase 2.

## Discussion

A major restructuring of perioperative care delivery is required to reduce cost while improving patient outcomes. ERAS pathways for CRS patients have been reported to have positive effects, but sustaining the gains made during the immediate post-implementation phase has been a challenge (Miller et al. 2014; Gillissen et al. 2015; Maessen et al. 2007; Stowers et al. 2015; Huang 2014; Geltzeiler et al. 2014; Paton et al. 2014; Hammond et al. 2014). The PSH model has been proposed and described but with minimal outcome data linked to the actual implementation of care redesign projects. In a prospective, observational (before and after) study of a healthcare redesign project, we investigated the effects of implementing a care pathway for CRS patients through a perioperative consult service at an academic tertiary care medical center. This implementation achieved reduced LOS and hospital costs, which resulted in an increased net financial margin to the hospital. We used control chart methodology prospectively to focus on an operational outcome, both to detect directional changes initially and to monitor ongoing performance. Most importantly, the changes from this care redesign project have met the goals of the IHI Triple Aim and have been sustained.

Similar to other reports on the implementation of ERAS pathways for CRS, we report a >20 % reduction in LOS without a change in readmission or reoperation rates. As with other reports, the reduction in LOS was associated with a high degree of use of the bundle components. But, it has been noted that LOS may be an inappropriate marker for measuring the initial success of an ERAS program, as initial gains may simply be due to organizational restructuring (Maessen et al. 2008). However, as described above, when we began this investigation, our surgical group had already standardized and streamlined many processes and the LOS at our institution was already in the top decile of NSQIP participating institutions for elective CRS. The implementation of an ERAS pathway through our PCS team advanced beyond this baseline level of high performance, and the overall result of this implementation is that we can now provide care for four CRS patients from decision to discharge with approximately the same hospital personnel and rooming costs as for three patients in the historical baseline group. (We confirmed the face validity of this assertion with our hospital operations executives.) In a full hospital with patients available to fill capacity created by operational improvements, reducing LOS is financially beneficial as more new patients can be given care (Krupka et al. 2012).

Additionally, beyond a sustained reduction in LOS and reduced cost per case, our new process of care delivery resulted in a high level of sustained adherence to ERAS pathway components, which has been reported to be difficult (Gillissen et al. 2015; Maessen et al. 2007). Our observed change is likely due to the implementation of ERAS pathway via the mechanism of our PCS team in close partnership with the colorectal surgeons, with a well-defined daily process. In our system of implementation, patient management is precise and personalized throughout the perioperative care arc, taking into account concerns of age, pain history, multi-system organ function, and current medications for co-morbidities, all while adhering to the goals of opioid avoidance, structured PONV prophylaxis, early re-feeding, and early ambulation in order to encourage faster functional recovery (Bryson 2015; Cerantola et al. 2013; Fayezizadeh et al. 2014; Fierens et al. 2012).

There are several limitations to our current study. The data presented come from a combination of data from our electronic medical record and administrative data, neither of which were collected specifically for the purpose reporting these results. The initial phase 0 patients were operated on during the planning phase of the project, and some ERAS components may have improved their LOS, diminishing the actual improvement we achieved relative to an earlier, true baseline. Our phase of results is currently over only 15 months. As the project is ongoing, monitoring will occur to assess whether these gains made by care redesign are permanently sustained in both process and outcome metrics. Finally, while the implementation of the ERAS care pathway via our PCS team was the only change made during this time, we cannot be certain which components of the care redesign are most important for achieving such gains. Attempts at sustaining LOS reductions with ERAS pathways alone have been shown to have difficulties and to often regress toward the historical mean (Gillissen et al. 2015; Maessen et al. 2007). It is known that ERAS pathways work, but the proper mode of sustained implementation is unknown. We have likely only demonstrated that our PCS team is one form of healthcare redesign that is successful. Other paradigms are likely possible.

### Practical lessons learned

While there are a myriad of lessons to be learned during any effort like the one that we have described, there are several practical points that we believe are important for any such implementation of healthcare redesign. First, we believe that *the key* aspect in all of our efforts has been the cooperative and collaborative efforts of the surgeons and anesthesiologists as physician leaders of the team. We truly approached this as a team with equal buy-in from the outset. If there is not 100 % commitment from both sides, initial or sustained success is unlikely. Second, proper messaging to all frontline care providers in the initial and ongoing planning, refinement, and implementation efforts is crucial. We did not do this as well as we should have initially. With all of the excitement to launch the ERAS pathway and our PCS, we did not take the time to message the coming changes properly to all involved. The principles and processes to be involved are reversing years of practice for all, and even decades of practice for some. Understanding the culture change that is needed to have this become the new way to care for colorectal surgical patients takes a lot of messaging as to the evidence and the “why.” As such, we have since added nurse anesthetist, resident, nursing, and pharmacist liaisons to the team to assist with ongoing dissemination of our results as well as educational reminders of the care processes throughout the institution.

Additionally, the team or department that attempts to do this may have to “do it on the margin” until local data can demonstrate local gains. This was true as we started our PCS, as we did not hire additional personnel at the outset. But, that quickly led us to have administrative leadership buy-in, which the final key ingredient. We report monthly updates to hospital leadership on bed-day savings relative to our historical baseline. Not only did this help us justify our funding request for two nurse practitioners to help with our postoperative rounding service, but it continues to keep the administrative leadership interested in scaling this to other services. Once the process is started, leveraging repetitive education and messaging to the frontline care providers and hospital leadership concerning the success and struggles of the service (as these will come) must be a part of the process or enthusiasm and effort will wane and regression to the prior state is likely to occur.

### Future directions

We are currently in the planning phase of our fourth PDSA cycle, which will include a new educational initiative and tool for patient engagement concerning goal-directed, rather than time-based discharge. Additionally, our model and methodology were built to be scalable, and we have recently expanded the perioperative consult service to cover weight reduction, complex ventral hernia repair, hepatobiliary/pancreatic, gynecologic oncology, and living donor nephrectomy surgical populations. This has been accomplished using the same physician personnel resources and care redesign process via the PCS. Future plans are underway to expand to five additional surgical populations within the next 6 months (King et al. 2015). Based on the economics of this project (liberated bed-days and reduced costs), hospital administration has given support for two additional nurse practitioners to staff these planned expansions. Throughout this process, we will continue to monitor the outcomes across all surgical populations cared for by the PCS in order to evaluate whether our care process and achieved gains remain stable.

## Conclusions

A care redesign project involving the implementation of a care pathway via a perioperative consult service with defined processes resulted in a significant and sustained reduction in LOS and hospital costs without an increase in readmissions or reoperations for colorectal surgery patients.

## Additional file

Additional file 1: Tables S1–S3 and Figure S1–S3. Mean pain scores by phase of implementation (Table S1), intraoperative and PACU intravenous fluid administration and urine output (Table S2), time to first oral intake and gastrointestinal output (Table S3), use of preoperative and intraoperative ERAS bundle components for multimodal analgesia before and after implementation of the ERAS pathway for colorectal patients (Figure S1), intraoperative and post-anesthesia care unit opioid use by phase (Figure S2), use of postoperative ERAS bundle components for multimodal analgesia before and after implementation of the ERAS pathway for colorectal patients (Figure S3). (PDF 478 kb)

## Abbreviations

APAP: acetaminophen
ASA: American Society of Anesthesiologists
BMI: body mass index
CI: confidence interval
CPT: current procedural terminology
CRS: colorectal surgical
dexameth: dexamethasone
ERAS: enhanced recovery after surgery
IHI: Institute for Healthcare Improvement
IRB: Institutional Review Board
IV: intravenous
LOS: length of stay
NSQIP: National Surgery Quality Improvement Project
OR: operating room
PACU: post-anesthesia care unit
PCA: patient-controlled analgesia
PCS: perioperative consult service
PDSA: plan-do-study-act
PEC: preoperative evaluation clinic
PONV: postoperative nausea and vomiting
PSH: perioperative surgical home
TAP: transversus abdominus plane block
TEC: thoracic epidural catheter
