# Supplementary material for: A perioperative consult service results in reduction in cost and length of stay for colorectal surgical patients: evidence from a healthcare redesign project
Source: Perioper Med (Lond). 2016 Feb 5;5:3. doi: 10.1186/s13741-016-0028-1 (PMC4743367; doi:10.1186/s13741-016-0028-1)
Supplement: Additional file 1: Tables S1–S3 and Figure S1–S3. — Mean pain scores by phase of implementation (Table S1), intraoperative and PACU intravenous fluid administration and urine output (Table S2), time to first oral intake and gastrointestinal output (Table S3), use of preoperative and intraoperative ERAS bundle components for multimodal analgesia before and after implementation of the ERAS pathway for colorectal patients (Figure S1), intraoperative and post-anesthesia care unit opioid use by phase (Figure S2), use of postoperative ERAS bundle components for multimodal analgesia before and after implementation of the ERAS pathway for colorectal patients (Figure S3). (PDF 478 kb) [file 13741_2016_28_MOESM1_ESM.pdf]

Additional files

Table S1. Mean Pain Scores by Phase of Implementation

Table S2. Intraoperative and PACU Intravenous Fluid Administration and Urine Output

Table S3. Time to First Oral Intake and Gastrointestinal Output

Figure S1. Use of preoperative and intraoperative ERAS bundle components for multimodal analgesia before and after implementation of the ERAS pathway for colorectal patients

Figure S2. Intraoperative and post-anesthesia care unit opioid use by phase

Figure S3. Use of postoperative ERAS bundle components for multimodal analgesia before and after implementation of the ERAS pathway for colorectal patients

Table S1

Mean Pain Scores by Phase of Implementation						
	Phase 0 (N=179)	Phase 1 (N=124)	Phase 2 (N=241)	P		
				0 v. 1	1 v. 2	0 v. 2
Postoperative Day 0	5.15±2.29	4.84±2.29	4.74±2.16	0.25	0.90	0.12
Postoperative Day 1	4.58±1.82	4.48±2.15	4.46±2.10	0.74	0.91	0.87
Postoperative Day 2	4.31±1.85	4.17±1.94	4.36±1.99	0.74	0.40	0.51

Data as Mean±SD.

Table S2

Intraoperative and PACU Intravenous Fluid Administration and Urine Output						
	Phase 0 (N=179)	Phase 1 (N=124)	Phase 2 (N=241)	P		
				0 v. 1	1 v. 2	0 v. 2
Intraoperative IV Fluid (mL)	2170±994	1740±901	1721±1020	<0.01	0.50	<0.01
Intraoperative Urine Output (mL)	270±217	234±178	242±186	0.36	0.7	0.47
PACU IV Fluid (mL)	781±639	652±673	653±893	<0.01	0.11	<0.01
PACU Urine Output (mL)	362±370	365±377	357±391	0.51	0.68	0.19

PACU = Post-Anesthesia Care Unit; IV = intravenous; Data as Mean±SD

Table S3

Time to First Oral Intake and Gastrointestinal Output[a]						
	Phase 0 (N=179)	Phase 1 (N=124)	Phase 2 (N=241)	P		
				0 v. 1	1 v. 2	0 v. 2
Hours to first PO intake (liquid)	9±6	8±5	8±6	<0.01	0.69	<0.01
Hours to first meal	44±38	44±32	35±25	0.93	<0.01	<0.01
Hours to first stool output[b]	30±25	24±16	22±18	0.03	0.33	<0.001

[a]all variables timed from the time patient left the operating room; [b]via rectum or ostomy; Data as mean±SD

Figure Legends

Figure S1: Implementation of preoperative and intraoperative ERAS pathway components for multimodal analgesia before and after implementation of the ERAS pathway for colorectal surgical patients. There was a significant increase in use of all components, in Phase 1 and 2 compared to Phase 0, except for ondansetron, which already had a high rate of use. Use of ketorolac, APAP, and gabapentin did not reach 100%. [ERAS = Enhanced Recovery After Surgery; TEC = Thoracic Epidural Catheter; TAP = transversus abdominus plane block; APAP = acetaminophen; Dexameth = dexamethasone]

Figure S2: Intraoperative and PACU opioid use by phase in morphine equivalents. There was a significant decrease in both intraoperative and PACU use of opioids in Phases 1 and 2 compared to Phase 0, with >80% reduction in intraoperative opioid use and >50% reduction in PACU opioid use. [PACU = Post-Anesthesia Care Unit; 95CI = 95th percentile Confidence Interval]

Figure S3: Utilization of postoperative ERAS pathway components for non-opioid, multimodal analgesia before and after implementation of the ERAS pathway for colorectal surgical patients. There was a significant increase in use of ketorolac, acetaminophen, and gabapentin in Phases 1 and 2 compared to Phase 0, with a significant decrease in PCA usage and nausea for Phases 1 and 2 compared to Phase 0. [PCA = patient-controlled analgesia; TEC = Thoracic Epidural Catheter; TAP = transversus abdominus plane block; APAP = acetaminophen; Dexameth = dexamethasone]

Figure S1

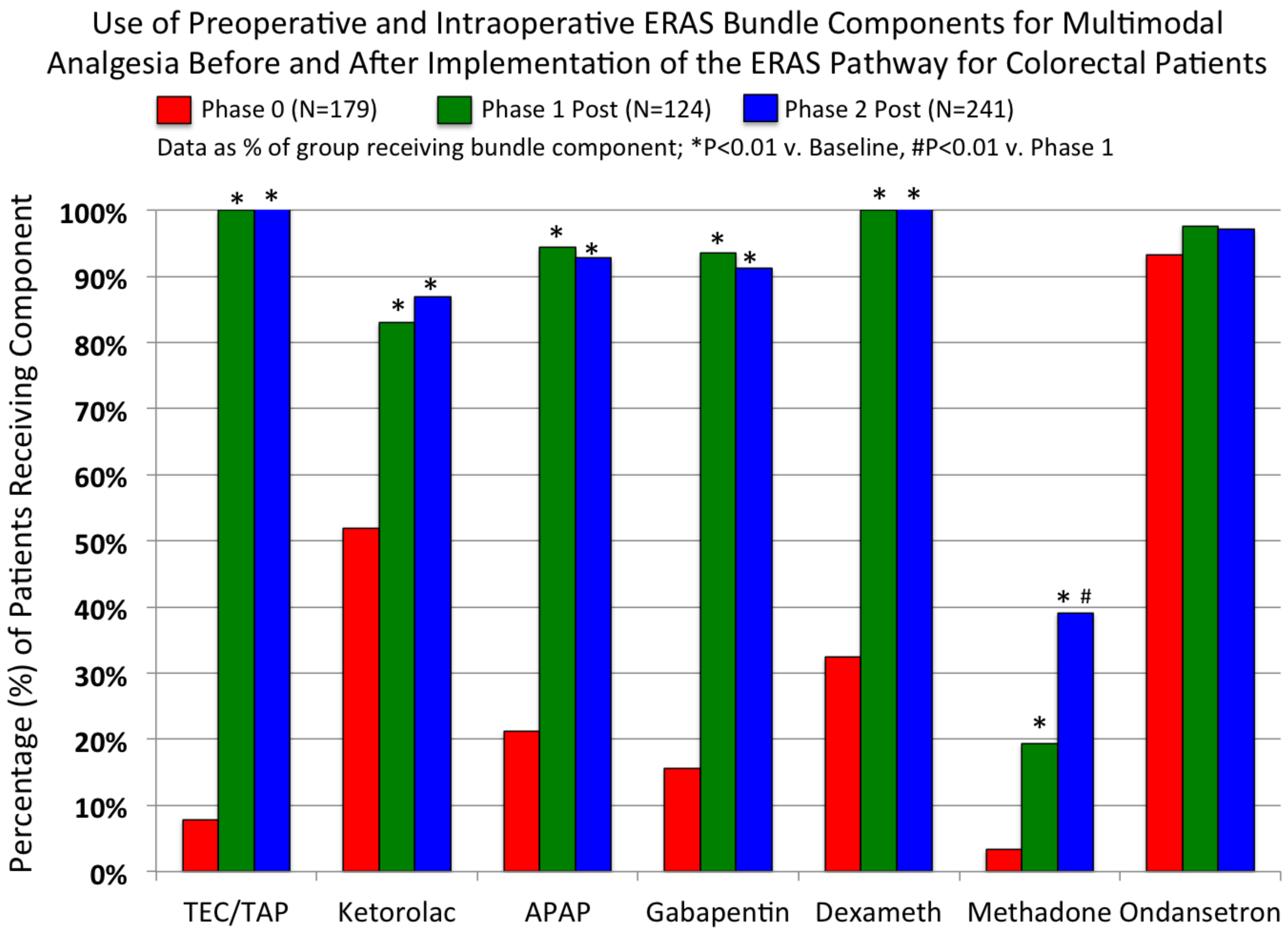
Use of Preoperative and Intraoperative ERAS Bundle Components for Multimodal Analgesia Before and After Implementation of the ERAS Pathway for Colorectal Patients
Phase 0 (N=179)
Phase 1 Post (N=124)
Phase 2 Post (N=241)
Data as % of group receiving bundle component; *P<0.01 v. Baseline, #P<0.01 v. Phase 1
Percentage (%) of Patients Receiving Component
100%
90%
80%
70%
60%
50%
40%
30%
20%
10%
0%
TEC/TAP
Ketorolac
APAP
Gabapentin
Dexameth
Methadone
Ondansetron
*
#

Figure S2

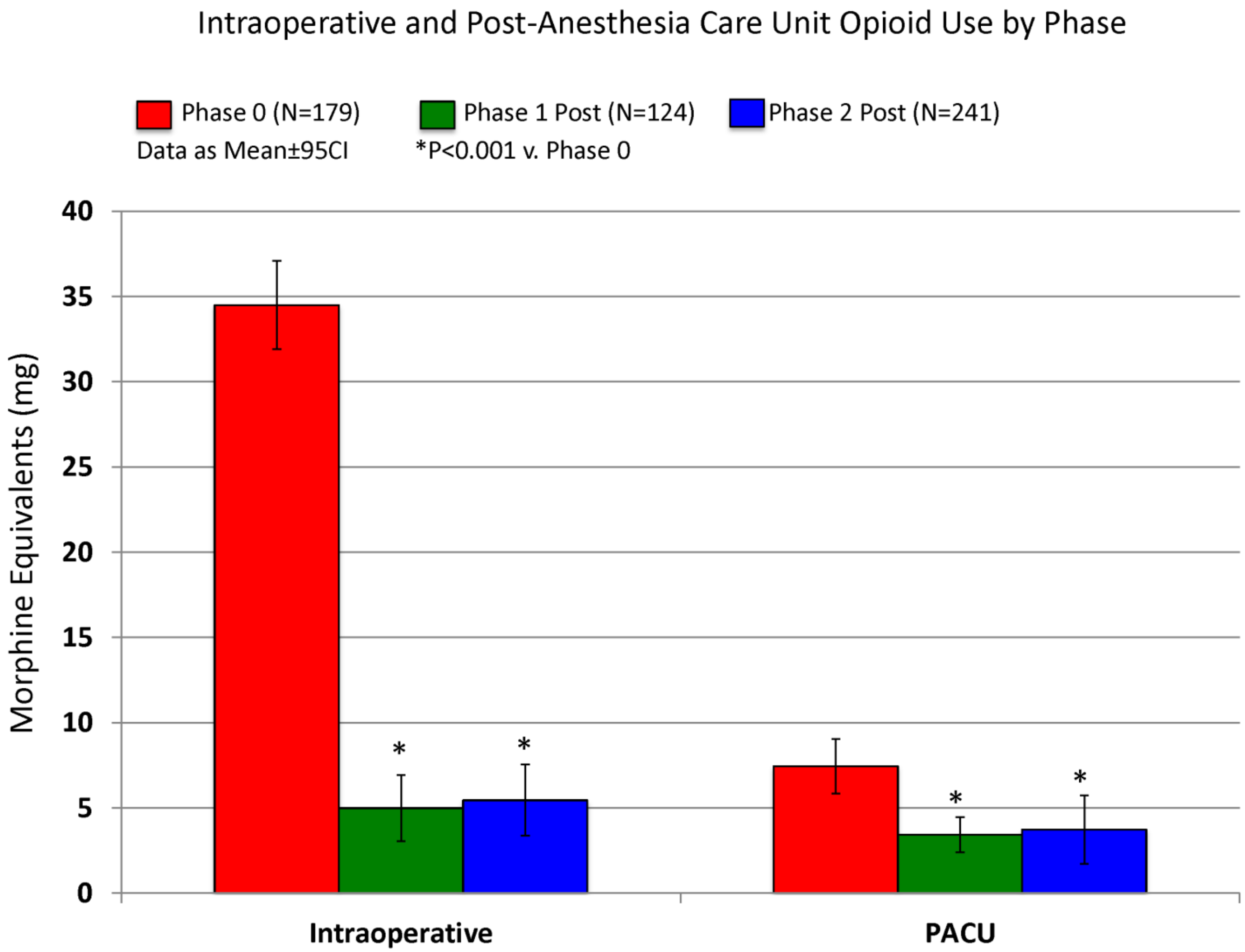
Intraoperative and Post-Anesthesia Care Unit Opioid Use by Phase
Phase 0 (N=179)
Phase 1 Post (N=124)
Phase 2 Post (N=241)
Data as Mean±95CI
*P<0.001 v. Phase 0
Morphine Equivalents (mg)
40
35
30
25
20
15
10
5
0
*
*
*
*
Intraoperative
PACU

Figure S3

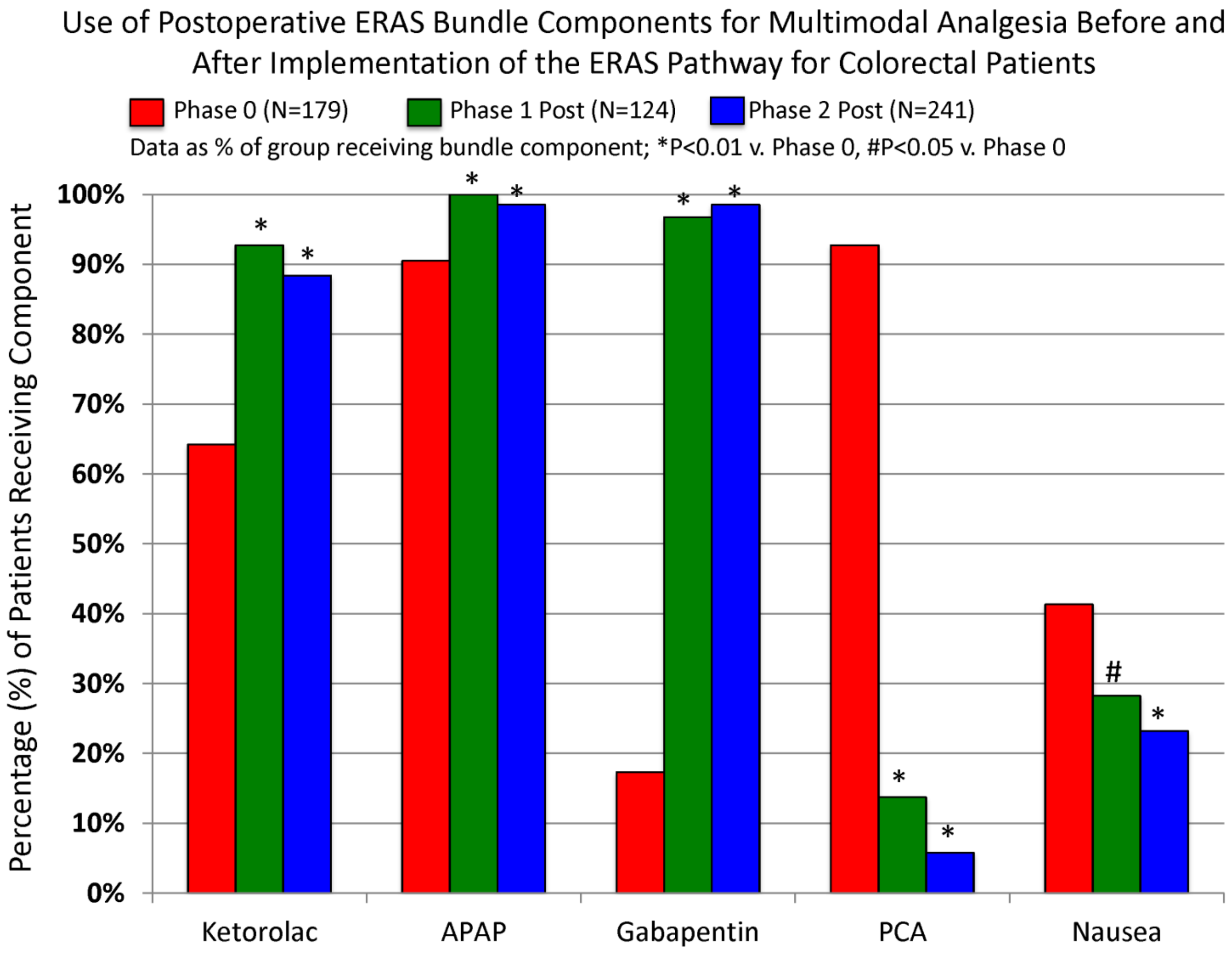

Use of Postoperative ERAS Bundle Components for Multimodal Analgesia Before and After Implementation of the ERAS Pathway for Colorectal Patients
Phase 0 (N=179)
Phase 1 Post (N=124)
Phase 2 Post (N=241)
Data as % of group receiving bundle component; *P<0.01 v. Phase 0, #P<0.05 v. Phase 0
Percentage (%) of Patients Receiving Component
100%
90%
80%
70%
60%
50%
40%
30%
20%
10%
0%
Ketorolac
APAP
Gabapentin
PCA
Nausea
